# The Relevance of Experimental Charge Density Analysis in Unraveling Noncovalent Interactions in Molecular Crystals

**DOI:** 10.3390/molecules27123690

**Published:** 2022-06-08

**Authors:** Sajesh P. Thomas, Amol G. Dikundwar, Sounak Sarkar, Mysore S. Pavan, Rumpa Pal, Venkatesha R. Hathwar, Tayur N. Guru Row

**Affiliations:** 1 Department of Chemistry, Indian Institute of Technology Delhi, New Delhi 110016, India; sajesh@iitd.ac.in; 2 Department of Pharmaceutical Analysis, National Institute of Pharmaceutical Education and Research (NIPER) Hyderabad, Hyderabad 500037, India; amol.dikundwar@niperhyd.ac.in; 3 Department of Chemistry, Aarhus University, Langelandsgade 140, 8000 Aarhus, Denmark; sarkar.sounak009@gmail.com; 4 Analytical Research and Development, Biocon Bristol Myers Squibb Research and Development Center, Syngene International Limited, Bangalore 560099, India; mspavanchem@gmail.com; 5 Faculty of Pure and Applied Sciences, University of Tsukuba, Tsukuba 305-8571, Japan; rumpa.sscu@gmail.com; 6 School of Physical and Applied Sciences, Goa University, Panaji 403206, India; vhathwar@unigoa.ac.in; 7 Solid State and Structural Chemistry Unit, Indian Institute of Science, Bangalore 560012, India

**Keywords:** non-covalent interactions, electron density, qtaim, multipole modelling, crystal

## Abstract

The work carried out by our research group over the last couple of decades in the context of quantitative crystal engineering involves the analysis of intermolecular interactions such as carbon (tetrel) bonding, pnicogen bonding, chalcogen bonding, and halogen bonding using experimental charge density methodology is reviewed. The focus is to extract electron density distribution in the intermolecular space and to obtain guidelines to evaluate the strength and directionality of such interactions towards the design of molecular crystals with desired properties. Following the early studies on halogen bonding interactions, several “sigma-hole” interaction types with similar electrostatic origins have been explored in recent times for their strength, origin, and structural consequences. These include interactions such as carbon (tetrel) bonding, pnicogen bonding, chalcogen bonding, and halogen bonding. Experimental X-ray charge density analysis has proved to be a powerful tool in unraveling the strength and electronic origin of such interactions, providing insights beyond the theoretical estimates from gas-phase molecular dimer calculations. In this mini-review, we outline some selected contributions from the X-ray charge density studies to the field of non-covalent interactions (NCIs) involving elements of the groups 14–17 of the periodic table. Quantitative insights into the nature of these interactions obtained from the experimental electron density distribution and subsequent topological analysis by the quantum theory of atoms in molecules (QTAIM) have been discussed. A few notable examples of weak interactions have been presented in terms of their experimental charge density features. These examples reveal not only the strength and beauty of X-ray charge density multipole modeling as an advanced structural chemistry tool but also its utility in providing experimental benchmarks for the theoretical studies of weak interactions in crystals.

## 1. Introduction

The question of what happens to an atom when it participates in the formation of a molecule and what happens to a molecule when it is put into molecular crystalline forms has been a central dogma, and answers were sought from various branches of science and philosophy over the last several decades. After it was evident that X-rays are scattered by the electrons of the constituent atom, ions, or molecules, mapping of the distribution of electron density in crystals as a consequence was imminent. However, the success of an ambitious experimental venture such as mapping accurate charge density distributions and their topology was realized only during the last few decades after significant technological advances in both data acquisition and computation. Charge density analysis occupies a prominent position in crystallography, particularly based on the pioneering work by Coppens, Stewart, Hirshfeld, and several other prominent investigators [1,2,3,4,5]. There are several review articles describing the methodology for data collection, analysis, and modeling of experimental electron density and indeed recent articles also bring in the strength and shortcomings of this technique [6,7]. In the context of structural chemistry, experiments were designed to evaluate the electron density distribution in a given molecule. The work performed in the Coppens group stands testimony to this approach with the calculation of the so-called “deformation density maps”. These maps facilitated the estimation of the extent of polarization in a covalent bond, the electrostatic field generated by the molecules or ions in a unit cell. However, on realizing the effect of thermal motion and the limitations due to the resolution of the data will restrain the quantification, the concept of static deformation density was invoked to ascertain the finer aspects in crystalline space. The use of the multipolar model defined a standard protocol and least-squares refinement of the parameters clearly produced interpretable deformation maps that could be quantified [2,3]. This led to a wide range of applications in probing unusual chemical bonding situations including intermolecular interactions. Currently, the methodology has become more feasible within laboratory X-ray sources and experimental conditions, opening remarkable possibilities for exploring various chemical bonding features in various classes of compounds. Aspherical modeling of atoms using multipole formalism against an accurate high-resolution X-ray diffraction data in combination with the application of the concept of “atoms in molecules” (AIM) methodology proposed by Richard Bader provides a quantitative assessment of the topological properties of various bonding features in crystals.

The recognition of the presence of noncovalent interactions, which invoked the concept of chemical bonding by J. D. van der Waals in his doctoral thesis in 1873, heralded the quest to understand these so-called “dispersion forces” [8]. Indeed, these forces in recent times have been recognized to have originated from quantum mechanics due to various electrostatic interactions between regions of different electronic charge densities. It is becoming increasingly apparent that the electronic signature of the condensed matter lies in both qualitative and quantitative understanding of van der Waal forces. Mapping of charge density features in intra- and intermolecular space is helpful in understanding and describing the binding properties in molecular systems and providing insights into their material properties.

The advent of crystal engineering principles recognizing the relevance of intermolecular interactions in molecular assemblies kindled the interest to extend the methodology to obtain insights into intermolecular interactions including strong and weak H-bonds [9]. This resulted in several applications of the charge density multipole method to crystal engineering problems, and a large number of publications emerged, particularly exploring weak and strong hydrogen bonds and their directional preferences explored based on the AIM methodology [10].

**Figure 1:**
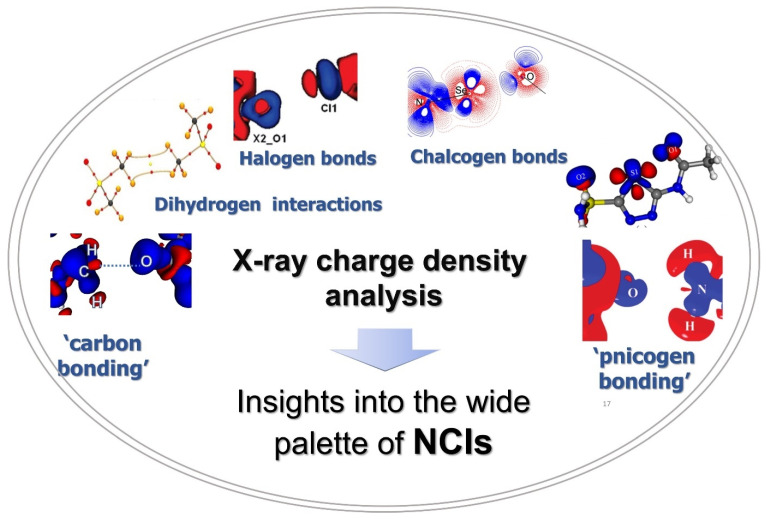
The wide palette of noncovalent interactions explored using X-ray charge density analysis.

In this mini-review, we describe charge density studies on weak interactions, other than hydrogen bonds—the highly directional non-bonded contacts like halogen, pnicogen, chalcogen, and carbon bonding interactions with specific examples which have been studied in our group in the last few years. Specifically, highlights of non-classical weak interactions involving group 14–17 elements are presented covering their nature, strength, and electrostatic origin (Figure 1). Applications of charge density studies in the field of crystal engineering and pharmaceuticals are briefed with specific examples. Further, a futuristic viewpoint for such studies is surmised invoking the methods which seek to overcome the deficiencies in the experimental charge density approach.

Due to the anisotropic packing of molecules or atoms in crystals, the potential field is never spherically symmetric, and hence approximating the overall crystal electron density as a sum of spherical atomic densities of constituent atoms may not be a valid consideration. To account for the aspherical distribution of electron density due to chemical bonding, Hansen and Coppens formulated the nucleus-centered finite multipole expansion of ρ [2]. In this formalism, the atomic electron density ρ is divided into three components

$$\rho_{\mathrm{at}}\left(r\right)=P_{c}\rho_{c}\left(r\right)+P_{\nu }\kappa^{3}\rho_{v}\left(\kappa r\right)+\overset{l_{\max}}{\underset{l=0}{\sum }}\kappa^{\prime 3}R_{l}\left(r\right)\overset{l}{\underset{m=0}{\sum }}P_{lm}\pm d_{lm}\pm \left(\vartheta ,\varphi \right)\;$$

ρ_c_ and ρ_v_ represent the spherical atomic scattering factors derived from ground-state Hartree–Fock wavefunctions, which are available in the form of databases. P_c_ and P_v_ are the spherical core and valence electron densities. P_v_, provides a rough estimation of the net atomic charge by q = N_v_ − P_v_, where N_v_ is the number of valence electrons in a free neutral atom. The third term in the summation is the aspherical valence density. The d_*lm*±_ are density normalized spherical harmonics of degree *l* and order *m*. P_*lm*±_ denotes multipole populations and *R_l_*(*r*) are the Slater type radial functions. The coefficients κ and κ′ represent the contraction–expansion of spherical and multipolar valence densities, respectively. The radial function *R_l_* in the deformation valence density is based on single-zeta Slater-type orbitals with energy-optimized exponents *ξ* taken from valence orbital wavefunction calculations.

$$R_{l}\left(r\right)=\frac{\xi^{n_{l}+3}}{\left(n_{l}+2\right)!}{\left(r\right)}^{n(l)}\exp\left(-\xi_{l}\;r\right)$$

The multipole population parameters P_*lm*±_, the expansion–contraction parameters k and k_0_ for the radial parts of the electron densities, positional parameters, and ADPs are refined against the experimental diffraction data using the least-squares refinement protocol. Several least-squares refinement packages have been developed to model electron density based on Hansen and Coppen’s multipole formalism by several groups. However, the most widely used packages are XD and MoPro for the modeling of electron density distribution [11,12]. Beyond pure geometrical considerations, the aspherical multipole modeling of electron density serves as a unique source of chemical bonding information that can be obtained experimentally.

Multipole modeling of high-resolution X-ray data can be achieved to efficiently deconvolute the thermal motion and the electron density. The correctness and reliability of the model are examined by the Hirshfeld rigid body test, residual density, and fractal dimension distribution of residual density [4,13]. Hirshfeld’s rigid bond test is one important criterion to assess the physical significance of the thermal parameters included in the multipole model. This test assumes that the chemical bond is rigid with respect to vibrational motion. If z^2^_A,B_ denotes the mean square displacement amplitude of atom A in the direction of atom B, for the two covalently bonded atoms,

Δ_A,B_ = z^2^_A,B_ − z^2^_B,A_ = 0

For a good quality multipole model, the anisotropic displacement parameters should qualify the criteria of the rigid bond test. If some of the bonds in the molecule do not fulfill the rigid bond postulate, it may be implied that the modeling is not complete and further structural improvements are required. As per the Hirshfeld test criteria, ∆_A,B_ is required to be lower than 0.001 Å^2^, least as for pair of atoms like carbon and those heavier than carbon. This is considered one of the most critical tests for the experimental electron density model to be reliable.

Another important test for a successful model, and to ensure the quality of the fit, is to inspect the residual electron density which gives a direct space representation of the extent to which the model accounts for the observed electron density. The difference in total electron density distribution ((∆ρbetween the observed and calculated electron density is the “residual density”, which represents the inadequacy in the fitted multipole model. A featureless residual map is one of the necessary conditions for the adequacy of a model; however, knowledge about its distribution in the unit cell is important for the validation of the model. Fractal dimension plots give information on the amount of residual density present along with its spatial distribution, i.e., the extent to which the distribution is featureless. The residual density features on the map indicate noise in the experimental data and also hint toward modeling shortcomings, suggesting the need for further improvements to the model. The parabolic shape of the fractal distribution indicates the presence of Gaussian noise in the residual density and provides a benchmark for improving the model to be refined further when fractal distribution deviates from this characteristic shape possibly due to various systematic errors [14].

## 2. Understanding Noncovalent Interactions in Terms of Descriptors from X-ray Charge Density Analysis

Here, we describe different classes of NCIs in molecular crystals, explored using the descriptors derived from X-ray charge density analysis. As most of the classical hydrogen bonds and weak hydrogen bonds are well explored for their electronic characteristics, here the focus is on the newer classes of NCIs that gained prominence over the last two decades.

### 2.1. Halogen Bonds

**Figure 2:**
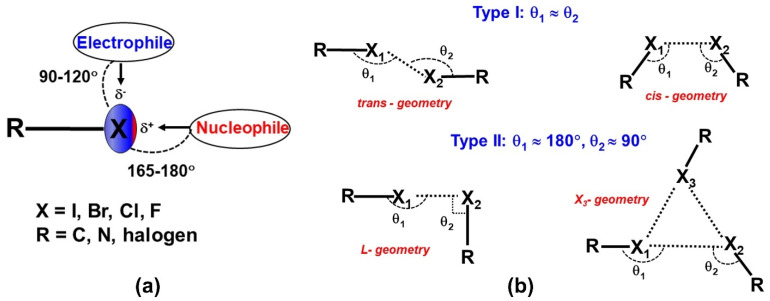
(**a**) Schematic representation of a typical halogen bond (**b**) Schematic representation of different geometries of X∙∙∙X interactions, where X is a halogen.

A halogen bond (XB) is a non-covalent interaction between a halogen atom and a nucleophile in a supramolecular assembly. Thus, the nature of halogen bonds is similar to that of the hydrogen bonds, where a polarized halogen atom occupies the position of the hydrogen atom as an electron density acceptor in the formation of Lewis acid-base pairs [15]. A halogen bond is defined as R–*X*∙∙∙*D*, where *X* is a halogen atom with an electrophilic region (Lewis acid), and *R* is a covalently bonded group to *X* and *D* is a nucleophile that acts as a halogen bond acceptor (Lewis base). The IUPAC definition of the halogen bond is, “A halogen bond occurs when there is evidence of a net attractive interaction between an electrophilic region associated with a halogen atom in a molecular entity and a nucleophilic region in another, or the same, molecular entity” [16]. Indeed, the XB is characterized by an anisotropic electron density (ED) distribution over covalently bound halogen (X) atoms such that the ED is depleted along with the R–X bond and resulting in the accumulation of ED on its sides (Figure 2a). Hence, a positive electrostatic potential (ESP) region is generated along with the covalent R–X bond and acts as the Lewis acid center for bonding. This effect is known as the “polar flattening effect” [17]. Hence, the covalently bonded halogen atom simultaneously can act as either Lewis acid or Lewis base. The positive electrostatic potential region along the R–X bond is also referred to as a σ-hole [18]. The formation of such positive potential regions along the R–X bond is caused by the half-filled p-orbital on the halogen atom, which creates an electron deficiency region in the outer lobe of that p-orbital. The σ-hole feature is observed not only in halogens but also in the elements of the groups 14–16 in the periodic table. Thus, the subsequent electrostatic attractive interaction by the σ-hole in the packing of molecules is also known as σ-hole bonding [19]. Among the halogens, the polar flattening (σ-hole) effect is enhanced in the order F < Cl < Br < I such that the F-atom has minute σ-hole features. The σ-hole is also responsible for the directionality of halogen bonds such that the nucleophiles approach the halogen atom in a straight line along with the R–*X* bond, whereas electrophiles approach it at the perpendicular direction of the R–*X* bond in most of the cases (Figure 2a). The σ-hole concept has helped to visualize the interaction of electronegative halogens with that of nucleophiles in the crystal lattice. Recently, the formation of halogen bonds is extensively utilized in (i) designing organic functional materials with interesting physical properties, (ii) separation of enantiomers from the mixture, (iii) supramolecular self-assembly of liquid crystals, (iv) displaying different polymorphic modifications, and (v) in the synthesis of polymers. Indeed, halogen bonds have received significant importance in biology due to the selective binding of small molecules to receptors mediated through halogen bonds. Thus, halogen bonds have become an important class of interaction in understanding the reaction mechanisms and chemical reactivity in several organic, metal–organic and biological systems.

From a historical perspective, early structural studies by Hassel in several molecular complexes of dihalogens with electron donor organic molecules reported the features of halogen interactions and described them as “halogen bridged molecules”. Among them, the molecular complex of bromine molecule with 1,4 dioxane displayed an infinite chain of Br∙∙∙O interactions in the crystal [20]. Afterward, the angular preference of interactions involving different halogens was systematically analyzed using the statistical analysis of reported structures in the Cambridge Structural Database (CSD) [21]. Indeed, the R–X···X–R interactions are characterized by three geometrical parameters, *R*_ij_ = X···X, two angles θ_1_ = R–X···X, and θ_2_ = X···X–R. The interaction geometries with θ_1_ ≅ θ_2_ are classified as type I interactions, whereas interactions with θ_1_ ≅ 180° and θ_2_ ≅ 90° belong to type II interactions [22]. Additionally, type I interactions are further categorized into ‘*cis*’ and ‘*trans*’ geometries based on the directionality of participating halogens in the interaction [23]. In most cases, ‘*trans*’ geometry occurs across the center of inversion symmetry whereas ‘*cis*’ is often found across ‘*2-fold*’ symmetry. The *L* and *X*_3_-synthon geometries are resulting in type II interactions (Figure 2b) [23]. The strength of the X∙∙∙X interactions have found to decrease in the order, I···I > Br···Br > Cl···Cl > F···F. Indeed, the strength of X∙∙∙X interactions is related to the polarization effects, which increase in the order, F < Cl < Br < I for molecular crystals.

**Figure 3:**
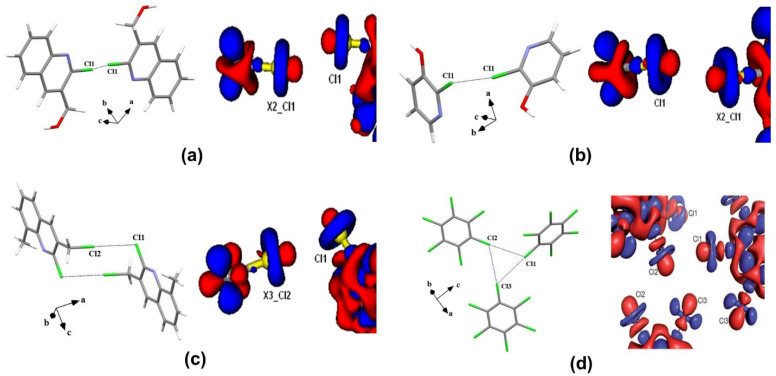
The 3D deformation density maps of Cl∙∙∙Cl interactions in (**a**) *cis*-geometry (**b**) *trans*-geometry (**c**) *L*-geometry and (**d**) *X*_3_-geometry. Adopted from references [23,26].

**Figure 4:**
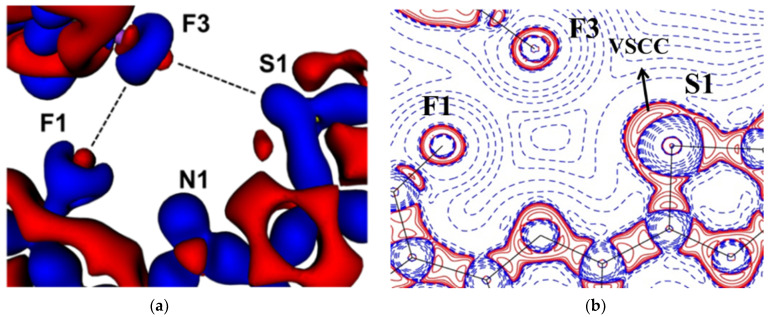
(**a**) Deformation density (Δρ(r)) (**b**) Laplacian (∇^2^ρ(r)) maps in the F···F and S···F interaction region. Δρ(r) isosurfaces are drawn at ±0.05 eÅ^−3^. ∇^2^ρ(r) (eÅ^−5^) drawn in logarithmic contours. (Figures adopted from [28]).

An early attempt to experimentally determine the ED distribution of halogens was performed by Stevens using high-resolution X-ray data by solid molecular chlorine crystals [24]. However, the quantitative information about the topology of ED distribution in the covalent bonding and intermolecular regions of solid molecular chlorine was later determined by Tsirelson et al. using QTAIM [25]. The attractive non-covalent nature of Cl∙∙∙Cl interactions was demonstrated by the charge concentration (CC) and charge depletion (CD) regions in Laplacian maps and bond paths between interacting chlorines. The topological properties of the ED further supported the attractive nature of halogen interactions in both experimentally and theoretically obtained EDs. Later, Bui et al. explored the nature of Cl∙∙∙Cl interactions in the *X*_3_-geometry of C_6_Cl_6_ molecule [26]. The multipole modeling of X-ray data confirmed that the X∙∙∙X interactions are directional attractive electrostatic (δ +···δ−) interactions in the crystal structure (Figure 3). The ED and Laplacian values at the bond critical point (BCP) for Cl∙∙∙Cl interactions were in the range of 0.03 < ρ_BCP_ < 0.06 eÅ^−3^ and 0.3 < ∇^2^ρ_BCP_ < 0.6 eÅ^−5^, respectively, and suggesting that the strength of Cl∙∙∙Cl interactions were corresponding to that of weak hydrogen bonds. Indeed, the X_3_ geometry is resulting in the crystal structure due to a cooperative organization of three side-on type II interactions and experimentally supported earlier “bumps-in hollow” hypothesis for XBs (Figure 3d). In another study, type I ‘*cis*’ and ‘*trans*’ and type II Cl∙∙∙Cl interactions were subjected to both experimental and theoretical ED models in three different compounds, 2-chloro-3-quinolinyl methanol, 2-chloro-3-hydroxypyridine, and 2-chloro-3-chloromethyl-8-methylquinoline, respectively [23]. The topological properties of the ED listed in Table 1 (for the three types of Cl···Cl interactions) and Table 2 (various other interactions involving halogens) suggest a closed shell nature of interactions. Both type I ‘*cis*’ and ‘*trans*’ interactions are resulting due to the reduced repulsion by polar flattening effects whereas type II interactions are due to electrostatic (δ +···δ−) attractions by the CC and CD regions of the participating halogen atoms (Figure 3). Among the halogens, the participation of “organic fluorine” in intermolecular interactions and structure stabilization is always controversial mainly due to its small size, high electronegativity, and smaller polarizability of ED [27]. Even molecular electrostatic potential (MESP) obtained from high-level theoretical calculations does not show significant σ-hole features on the organic F-atom [18]. Interestingly, the ED model from the X-ray data depicted a tiny σ-hole region on the organic fluorine in 2-chloro-4-fluorobenzoic acid and 4-fluorobenzamide, where the C–Cl∙∙∙F–C and C–F∙∙∙F–C interactions are characterized, respectively [18]. In another ED study on pentafluorophenyl 2,2′-bithiazole, the cooperative role of C–F∙∙∙F–C and C–F∙∙∙S–C interactions involving organic fluorine in structure stabilization was demonstrated (Figure 4) [28]. Further, recent ED studies [29,30,31] further established the ‘σ-hole’ on organic fluorine in different chemical environments to establish XB involving fluorine as a realistic interaction in supramolecular assembly.

The ED studies on classical XBs like C–Cl∙∙∙O=C have supported the “bumps-in hollow” hypothesis of XBs such that a lone pair of oxygen is facing the CD region of the chlorine resulting in attractive interaction [33]. The study on a serious environmental pollutant, octachloronaphthalene, has evaluated both peri and intermolecular interactions to determine their effects on molecular conformation and the subsequent effect on aromaticity [36]. The steric hindrance by overcrowding as well as peri interactions is overcome by stabilizing intermolecular Cl∙∙∙Cl and Cl∙∙∙π interactions. The experimental quantification of intermolecular interactions involving heavier halogens like bromine and iodine is challenging due to absorption problems in the data. Even then, the experimental ED results from a laboratory X-ray data on Br∙∙∙Br [34,37], Br∙∙∙Cl [35,37], Br∙∙∙π [46], C–N∙∙∙Br [34,47], and C–I∙∙∙N [43,45,48], C–I∙∙∙O [43,44] XBs were remarkably comparable with those obtained from theoretical calculations. The true testament to the strength of halogen bonds involving bromine can be observed in the mentioned studies, where despite the lack of any strong hydrogen bond donors, the molecules crystallize as solids at room temperature, via only halogen bonding motifs. On the contrary, all molecules with halogen bonds involving fluorine are seldom solids at room temperature and are studied by in situ cryocrystallographic or high-pressure crystallographic techniques to obtain the molecule in a solid state [30]. In another study, the ED analysis on two isomeric compounds, 4-bromo-2-chloro benzoic acid (4Br) and 2-bromo-4-chlorobenzoic acid (2Br) could establish the role of triangular XB motif presence in 4Br to dictate the packing of molecules in solid solutions of 4Br and 2Br [37]. The ED study on the C–I∙∙∙N halogen bond in a molecular adduct of quinuclidine and iodobenzene provides one of the weakest halogen bond geometries, as the iodobenzene lacks electron-withdrawing group [49]. Even though, the N···I contact distance is the longest (2.9301 Å), the electron density in the BCP is 0.186(4) eÅ^−3^ and establishing it as a directional stabilizing interaction in the crystal structure. The ED results were further supported by 1D and 2D NOESY measurements.

**Figure 5:**
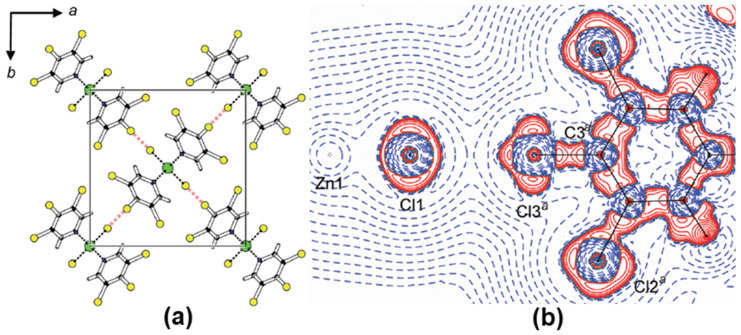
(**a**) The packing diagram and (**b**) the Laplacian map showing Cl∙∙∙Cl interactions in ZnCl_2_(3,4,5-trichloropyridine)_2_. Adopted from the reference [41].

The XBs in coordination polymers and metal complexes are subjected to the experimental ED studies to evaluate the nature of charge assisted XBs and compare their results with XBs in organic molecular compounds [44,50,51]. Indeed, the shortest Cl∙∙∙Cl interaction of distance 3.1912(6) Å was observed in ZnCl_2_(3,4,5-trichloropyridine)_2_ (Figure 5a). The ρ_BCP_ and ∇^2^ρ_BCP_ values for the Cl∙∙∙Cl interaction were 0.107(2) eÅ^−3^ and 1.102(4) eÅ^−5^, respectively, and both the values were significantly larger when compared to Cl∙∙∙Cl interactions in organic compounds. Further, this study experimentally demonstrated that the chlorine bonded to an electron-rich arene ligand acts as a nucleophile by donating an electron density towards the polarized chlorine coordinately bonded to the central Zn metal cation (Figure 5b). The experimental observations are recently further corroborated by several theoretical studies based on the topological analysis [52,53].

### 2.2. Chalcogen Bonds

**Figure 6:**
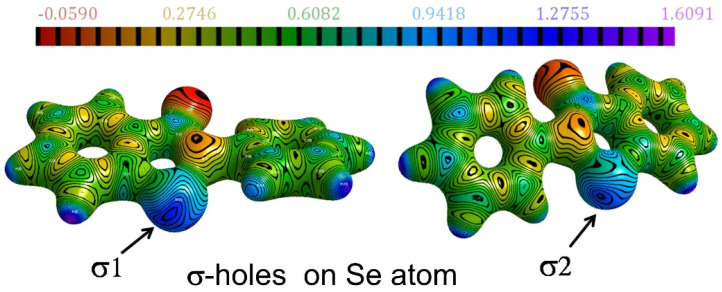
The σ-holes around the Se atom in *ebselen*, revealed on the ESP mapped on isoelectron density surface (ESP scale in e/Å, the electron density isosurface value =0.5 e/Å^3^). Adopted from the reference [48].

One of the earliest experimental charge density studies on the intermolecular chalcogen bonding (ChB) interactions and their ability to form robust supramolecular synthons was reported by Thomas and Row in the Twenty-Second Congress and General Assembly of the International Union of Crystallography (IUCr2011, Madrid) [54]. At that time, when halogen bonding was rapidly emerging as a new type of non-covalent interaction and finding its applications in crystal engineering and supramolecular chemistry, we reported the potential of organic selenium atom to form unusually short Se∙∙∙O chalcogen bonds in the polymorphs of the organoselenium antioxidant *ebselen* and its hydroxy derivative. Se∙∙∙O interactions observed in the series of crystal structures analyzed in our study represented some of the shortest intermolecular Se∙∙∙O chalcogen bonds known for crystalline organoselenium compounds [48]. The robustness and electronic features of the Se∙∙∙O chalcogen bonds in ebselen were revealed by the high-resolution X-ray charge density models and topological analysis. More importantly, the existence of dual σ-hole behavior around the Se atom was unraveled in the electrostatic potentials mapped on isoelectron density surfaces of *ebselen*—thus pointing to the potential of bifurcated chalcogen bonding in such molecules (Figure 6). Topological analysis of the Se-N and Se-C covalent bonds along with the Se∙∙∙O chalcogen bonds in *ebselen* and its hydroxy analog provided some fundamental insights into the Se-N bond cleavage mechanism involved in the drug action of this class of organoselenium antioxidants. The Laplacian profiles from experimental charge density linked the biological activity and low toxicity exhibited by *ebselen* to the polarized nature of Se-N bond and a highly covalent Se-C bond (See Table 3). In addition, we reported the FTIR spectral features of the Se∙∙∙O chalcogen bonds from solution state to solid crystalline state in *ebselen* further characterized this class of supramolecular recognition units.

Interestingly, the sulfur analog of this chalcogen bonded supramolecular synthons was observed and characterized by us recently in a series of multi-component crystals of the drug *riluzole*, in which two σ-hole regions were found around the S atom, which formed a variety of S∙∙∙O, S∙∙∙F, and S∙∙∙Cl chalcogen bonds [50]. ESP mapped on the Hirshfeld surface of *riluzole* showed regions corresponding to the σ-holes on the S atom, analogous to those found around the Se atom in *ebselen* analogs. We also found that the σ-hole regions around S atom visualized from the ESP plots corresponded to the LUMO (lowest unoccupied molecular orbital) density mapped onto the Hirshfeld surfaces of *riluzole* [50]. It should be mentioned in this context that the first experimental charge density study of the ChBs involving Se and O atoms was reported by Espinosa et al. [51] in which they characterized the ChBs as electrophilic–nucleophilic type interactions. They established the strength of Se∙∙∙O and Se∙∙∙Se chalcogen bonds (along with Se∙∙∙H interactions) found in the crystal structure of chalcogenophthalic anhydrides (Table 3), and that the directionality and geometrical preferences of the ChBs were driven by the CD∙∙∙CC nature of chalcogen bonds.

**Figure 7:**
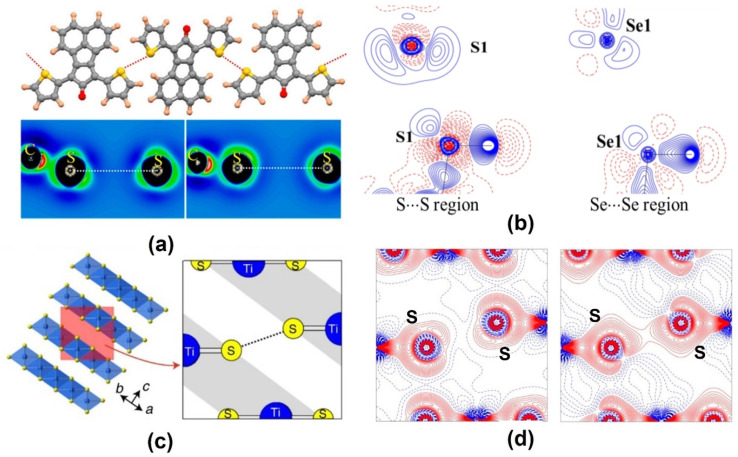
(**a**,**b**) S∙∙∙S and Se∙∙∙Se homo-chalcogen interactions explored using charge density multipole modeling (CDMM) method in a series of crystal structures. (**c**) Layered structure of TiS_2_ showing interlayer S∙∙∙S interaction. (**d**) Theoretical (left) versus experimental (right) deformation density maps for the interlayer S∙∙∙S interaction region—the overlapping density in the experimental map is notable. (Figures reproduced from references [55,56,57]).

Aside from the hetero-chalcogen interactions, a number of examples of S∙∙∙S and Se∙∙∙Se homo-chalcogen interactions have also been investigated using charge density multipole modeling. We have explored two different modes of the S∙∙∙S homo-chalcogen interactions in the donor−acceptor−donor structured organic conductor crystal of 7,9-di(thiophen-2-yl)-8H-cyclopenta[a]acenaphthylen-8-one (DTCPA), where both the CD∙∙∙CC and CC∙∙∙CC interaction modes were observed from the multipole charge density models obtained from the theoretical structure factors (Figure 7) [55].

Similarly, in the low-temperature crystal structures of the room temperature liquids thiophenol and selenophenol, we characterized the S∙∙∙S and Se∙∙∙Se homo-chalcogen interaction regions [56]. While the S∙∙∙S interaction in thiophenol was of the CD∙∙∙CC nature, such an electrostatic complementarity was not found for the S∙∙∙S interaction in selenophenol. An interesting example of the S∙∙∙S interactions in the layered structure of the material TiS_2_ was investigated by Iversen et al. recently [57]. Their experimental charge density models were found to be successful in bringing out the features of the interatomic S∙∙∙S interaction between the layers, which theoretical calculations failed to capture. The nature of these interlayer homochalcogen interactions was also found to be CC∙∙∙CC. In such examples, the crystal packing and the bind of layers along the crystallographic directions stabilized by S∙∙∙S interactions need to be understood in terms of dispersion forces as opposed to the electrostatic attraction. Table 3 shows bond properties of a collection of chalcogen bonds explored using charge density analysis. It may be noted that the same kind of interaction possesses different topological parameters in case of halogen interactions, such as F···F interaction, Br···Br interaction (Table 2) and chalcogen interactions S···S interactions in Table 3. This is a consequence of the variation of interaction distances, relative orientations of interacting atoms, and the electronic environment in different molecules.

**Table 3:** Topological parameters for noncovalent chalcogen interactions.

Interaction	*R*_ij_ (Å)	ρ (e Å^−3^)	∇^2^ρ (e Å^−5^)	V	|V|/G	Comment
Se∙∙∙O	2.5331	0.251	2.452	−84.7	1.12	Thomas, Row et al. [48]
Se-C	1.8842	1.03	0.50	−661.0	1.96	Thomas, Row et al. [48]
Se-N	1.8987	0.94	3.20	−592.7	1.74	Thomas, Row et al. [48]
Se∙∙∙O	3.355	0.049	0.62	−9.7	0.73	Espinosa et al. [51]
Se∙∙∙H	2.974	0.05	0.51	−8.9	0.78	Espinosa et al. [51]
Se∙∙∙Se	3.822	0.051	0.37	−7.7	0.87	Espinosa et al. [51]
S∙∙∙S	3.227	0.092	0.76	−18.6	0.95	Owczarzak et al. [58]
S∙∙∙S	3.365	0.083	0.34	−13.0	1.17	Owczarzak et al. [58]
S∙∙∙S	3.459	0.072	0.65	−13.7	0.87	Owczarzak et al. [58]
S∙∙∙S	3.443	0.086	0.691	−16.7	0.94	Iversen et al. [57]
S∙∙∙S	3.6291	0.071	0.547	−12.6	0.92	Bai, Row et al. [55]
S∙∙∙S	3.7927	0.042	0.376	−6.6	0.78	Bai, Row et al. [55]
S∙∙∙S	3.5837	0.07	0.6	−12.9	0.88	Thomas, Row et al. [56]
Se∙∙∙Se	3.7562	0.05	0.5	−8.8	0.78	Thomas, Row et al. [56]
S-H∙∙∙S	3.1078	0.02	0.4	−4.6	0.59	Thomas, Row et al. [56]
Se-H∙∙∙Se	3.0882	0.03	0.5	−6.3	0.64	Thomas, Row et al. [56]

**Figure 8:**
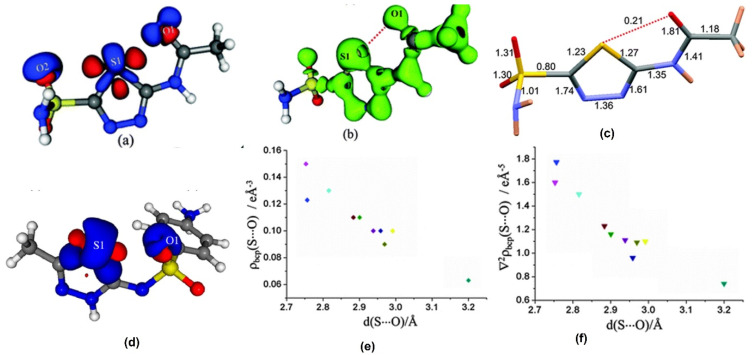
(**a**,**d**) The 3D deformation density maps reveal the CD∙∙∙CC nature of intramolecular S∙∙∙O ChBs in *acetazolamide* and sulfamethizole, respectively. (**b**) Laplacian isosurface showing the features corresponding to sigma-holes around S atoms, (**c**) bond orders corresponding to different bonds, and S∙∙∙O chalcogen bond in *acetazolamide*. (**e**,**f**) electron density and Laplacian at the BCPs of intramolecular S∙∙∙O chalcogen bonds derived from experimental charge density models reported in the literature. (Figures reproduced from references [59,60]).

As opposed to halogen bonds, the directionality of σ-holes around chalcogen atoms facilitate intramolecular NCIs as well. Among these, the intramolecular S∙∙∙O chalcogen bonds found in sulfa drugs are particularly interesting. In the sulfa drugs *acetazolamide* and in the salt of sulfamethizole, we characterized the experimental charge density features of the intramolecular S∙∙∙O chalcogen bonds which confirmed that these interactions are closed-shell type in nature (Figure 8) [59,60]. The electron density ρ (accumulation of density) and ∇^2^ρ (curvature of density distribution) values in the S∙∙∙O chalcogen bond regions were used to obtain the local kinetic energy density (G) and potential energy density (V) values. Table 4 shows topological parameters for the electron density distribution in the S∙∙∙O interaction region from a collection of reported examples for intramolecular S∙∙∙O chalcogen bonds based on experimental and computational studies. Notable among these examples is the detailed charge density study on the polymorphs of *sulfathiazole* by Farrugia et al. [61] in which the intramolecular S∙∙∙O chalcogen bond between the thiazole sulfur atom and oxygen in the sulfone group is consistently observed in all the five polymorphs indicating that these intramolecular interactions have the potential for molecular conformation locking. Our study on a structurally similar drug, sulfamethizole (in its sulfate salt form) also resulted in similar results indicating the conformation locking potential of intramolecular S∙∙∙O ChBs in competition with the relatively stronger N-H∙∙∙O hydrogen bonds. Such intramolecular motifs have an indirect but significant role in crystal packing, as they guide molecular confirmation. Studies on the series of sulfa drugs discussed here indicate that it is the interplay of intramolecular S∙∙∙O ChBs and the intermolecular HBs that dictate the conformations and crystal packing interactions.

### 2.3. Carbon Bonding, Pnicogen Bonding, and Hydrophobic Interactions

**Figure 9:**
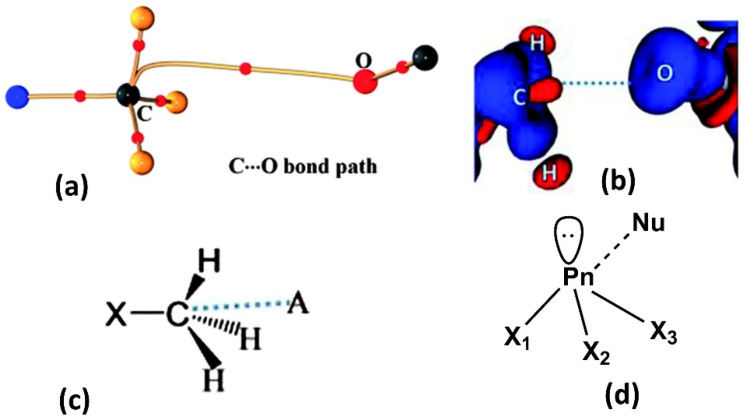
(**a**) Electron density bond path and (**b**) 3D deformation density maps revealing the nature of C∙∙∙O carbon bonding interaction. The Δρ iso-surfaces are drawn at ±0.02 e Å^−3^. (**c**) a general scheme diagram showing the geometry of carbon bonding at -CH_3_ atom with the acceptor atom A. (**d**) Schematic illustration of a pnicogen bonded motif between a Group VA (Pn) atom and a nucleophile (Nu). (Figures adopted from references [65,69]).

In addition to the predominant halogen bonds and chalcogen bonds, a new class of tetrel carbon bonding interaction attracted our attention, soon after Mani and Arunan’s work based on theoretical calculations [64] that “sp^3^” hybridized carbon atoms could also offer a σ-hole region (an electrophilic center) towards interactions with charge concentrated atoms such as oxygen. These non-covalent “carbon bonding” interactions exhibiting pseudo-hypervalent (pentavalent) carbon atoms were soon experimentally validated by our X-ray charge density study [65]. Our experimental charge density model showed bond paths from the nucleophilic O atom to the “sp^3^” carbon atom in the –CH_3_ group in the crystal structure of dimethylammonium 4-hydroxybenzoate, and unraveled its CD-CC nature (Figure 9a,b). Electron density topological parameters evaluated at the BCP of this C∙∙∙O carbon bonding interaction have been to be *ρ*_b_ = 0.03 e Å^−3^ and ∇^2^*ρ*_b_ = 0.6 e Å^−5^ for a bond path length, R_ij_ = 3.168 Å. This interaction motif was reminiscent of the nucleophilic attacking mode in the bimolecular substitution reaction (S_N_2) and hence could have some link to its supramolecular origin. Further, several detailed studies revealing the existence and ubiquity of these interactions in small model molecules and even in biomolecules were reported [66,67,68]. A recent computational study along with a protein data bank analysis by Biswal et al. showed that carbon bonding interactions are abundant in proteins and that they have a significant enthalpic contribution to the binding of nucleobases to proteins, hydrophobic interactions, and in the photodissociation mechanism of myoglobin [68].

Similar to the donor sites in halogen and chalogen bonding, members of the Group VA family (Pn = N, P, As, Sb, or Bi) are also characterized by a localized σ-hole. This σ-hole feature occurs along with the extensions of Pn–X covalent bonds. In the same manner, as halogen/chalcogen bonding, pnicogen bonding is formed when a nucleophilic (Nu) entity interacts favorably (lowering of free energy) with an electrophilic site or σ-hole of the pnicogen atom in the same or another molecule. Thus, a pnicogen atom can act as a multidentate pnicogen bond donor due to the availability of three potential sigma holes (Figure 9d). Compared to typical hydrogen bonds, pnicogen bonds are highly directional and sensitive to angular distortion like halogen bonds. The preference for a linear geometry X-P∙∙∙Nu, approaching 180° is mainly because of short-range exchange repulsion between same spin electron densities of pnicogen atom and pnicogen bond acceptor.

Hawkins et al. first observed P∙∙∙P intramolecular interactions in stereochemically active phosphanyl-ortho-carborane derivative from 13C NMR [70]. Along with these experimental observations, they also performed gas-phase computational studies on a series of phosphorous (III) dimers and concluded that non-bonding P∙∙∙P interactions in those dimers are probably due to the negative hyperconjugation of lone pair of electrons at one of the P center into the σ* antibonding orbital at the adjacent P molecule along P∙∙∙P axis [71]. Following this study, numerous theoretical research works were carried out to understand pnicogen interactions/bonding in P, As, Sb, containing compounds, etc. [72,73,74]. Typically, only the heavier pnicogen atoms are expected to form pnicogen bonds due to their high polarizability. Sb and Bi are used as an efficient pnicogen bonding donors for formation of supramolecular architectures in solution, solid state, catalysis, etc. [75,76,77]. In this context, the propensity of nitrogen, being the third most electronegative element with its extremely low polarizability, to act as a pnicogen bond donor remains questionable.

**Figure 10:**
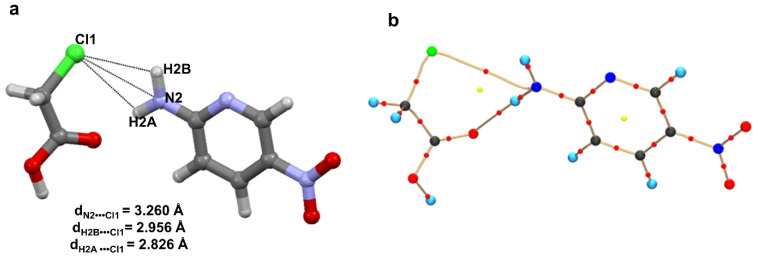
(**a**) Plausible N∙∙∙Cl interaction in the co-crystal of 2-amino-5-nitropyridine and chloroacetic acid. (**b**) Molecular Graph derived from experimental charge density model shows bond paths along with BCPs of the N∙∙∙Cl pnicogen bond and N-H∙∙∙O hydrogen bond in the structural motif (I) The yellow dots represents the (3,+1) ring critical points (RCP) while the red dots represent the (3,−1) BCPs. The curved segments signify the bond paths between atoms. Figure reproduced from reference [69].

This notion was challenged when a potential pnicogen bonded motif involving N as a donor was identified in a co-crystal of 2-amino-5-nitropyridine and chloroacetic acid [69]. The accurate electron density of this molecule was determined using 100 K high resolution (sinθ/λ = 1.08 Å^−1^) X-ray data based on multipole modeling. QTAIM analysis [10] on the multipole model confirmed a bond path between the electrophilic N and nucleophilic Cl atoms (Figure 10); hence, the first experimental evidence of pnicogen bonding in nitrogen. Low ρ value (~0.05 eÅ^−3^) and positive Laplacian values at the bond critical point classify this N∙∙∙Cl bonding as a weak closed-shell interaction similar to other weak intermolecular interactions such as type **II** F∙∙∙F [28], C∙∙∙O carbon bonding [65], CH_3_∙∙∙CH_3_ hydrophobic interaction [78]. Furthermore, this weak N∙∙∙Cl interaction is supported by strong H-bonding such as N-H∙∙∙O and O-H∙∙∙N.

**Figure 11:**
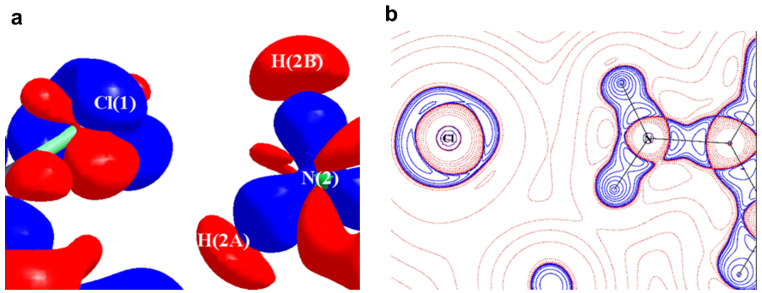
(**a**) The 3D static deformation density map drawn on isosurface of ± 0.08 eÅ-3. Blue regions indicate the charge concentration regions, while red regions indicate charge-depleted regions; (**b**) 2D plot of Laplacian map drawn at logarithmic scale. Blue solid lines and red dots represent positive and negative contours, respectively. (Figure reproduced from reference [69]).

The experimental static deformation densities, ρ_def_ = ρ_model_ − ρ_IAM_, reveal interesting details about the N∙∙∙Cl pnicogen bonds (Figure 11a). The deformation map shows that the lone pairs of the Cl atom (blue lumps) face the charge-depleted site or σ-hole (half red disc) at the nitrogen atom (Figure 11b). From the 2D Laplacian maps, it can be observed that lone pairs in the valence shell charge concentration (VSCC) region of the Cl atom point toward the charge depleted region of the N atom (Figure 11b).

**Figure 12:**
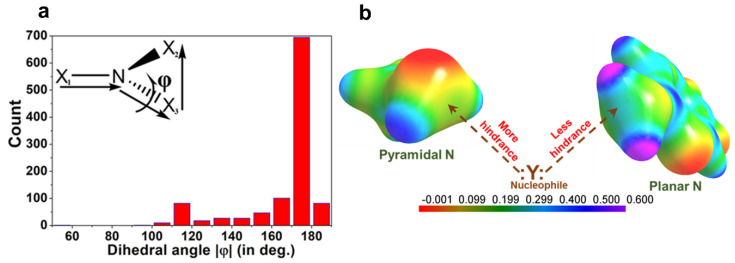
(**a**) Histogram of dihedral angle Φ distribution in the trivalent NX_1×2×3_ molecule found in 972 crystal structures containing pnicogen bond motif. (**b**) Comparison of 3D electrostatic potential maps (ESP) between methylamine (Pyramidal N) and 2-amino-5-nitropyridine (Planar N). The σ-hole region in the N atoms of both molecules is shown by broken arrows. ESP maps are mapped on isoelectron density surface (at 0.5eÅ^−3^). Gas-phase DFT calculations of the ESP maps are performed with a M062X functional and 6–311+g(d) basis set. Electrostatic potential scale is from—2.6 kJmol^−1^ to 15.8 kJmol^−1^. (Figure reproduced from reference [69]).

A detailed search was performed in the Cambridge Structural Database (CSD, version 5.39, November 2017) with the two geometric constraints: (a) the distance between the nucleophilic X atom and pnicogen bond donor nitrogen atom is less or equal to the sum of the van der Waals radii, (b) the angle ∠X − N∙∙∙Y (nucleophile) is restricted between 165°–180°. This search resulted in 972 crystal structures. The histogram of the dihedral angle Φ (X1-N-X3-X2) distribution for these structures shows that the probability of pnicogen bond formation is higher when the nitrogen moiety is planar (X1-N-X2-X3; inset in Figure 12a). Due to electron delocalization between N center and neighboring atoms, the repulsion between corresponding lone-pair and bond pair gets reduced in planar N moiety as compared to pyramidal N. Hence, the widening of ∠X2-N-X3 bond angles (~107° to 120°) from pyramidal to trigonal planar geometry favors the interaction between the nucleophile and the σ-hole on the nitrogen atom. Alternatively, it could be argued that σ-hole region in a planar N (2-amino-5-nitropyridine) is more electropositive than a pyramidal N center (methylamine) (Figure 12b).

**Figure 13:**
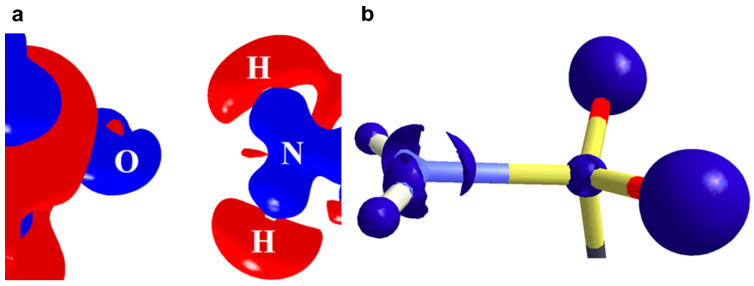
(**a**) Experimental 3D deformation density of the N∙∙∙O pnicogen bond interaction region drawn at the intervals of ±0.08 e·Å^−3^. Blue represents charge concentration (CC) and red represents charge depletion (CD). (**b**) Experimental 3D Laplacian isosurfaces around the N atom of sulphonamide group plotted at −40 eÅ^−5^ contour level. Figure reproduced from reference [63].

We further extended our charge density analysis of pnicogen bonding on the metastable polymorphic form of *acetazolamide* drug [63]. From the experimental electron density analysis, a weak N∙∙∙O intermolecular pnicogen bonding is observed. The 3D Laplacian map shows an almost negligible lone pair feature on the planar N center of the sulphonamide moiety (Figure 13). Interestingly, the pyramidal N atom in the thermodynamic polymorphic form of *acetazolamide* does not engage in any form of σ-hole based interaction.

**Figure 14:**
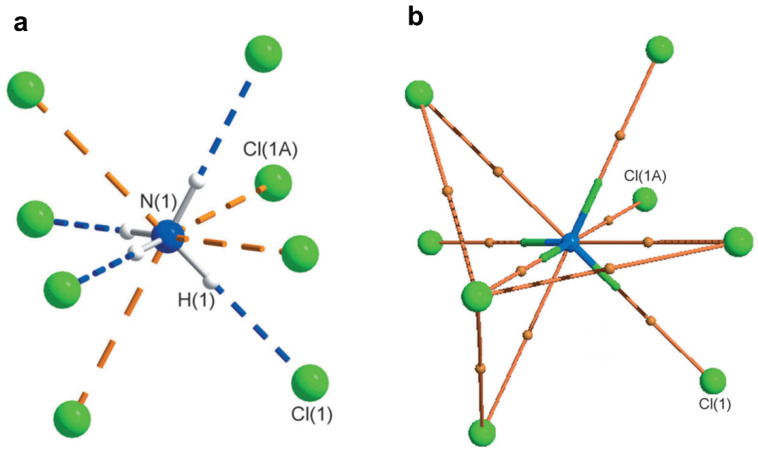
(**a**) Ammonium cation environment in NH_4_Cl (thermal ellipsoids for atoms drawn at probability of 90%); (**b**) bond paths between the two interacting atoms (ions) are shown by orange lines and bond critical points are depicted as orange spheres. Reprinted with permission from reference [80]. Copyright 2014, John Wiley and Sons [79].

Lyssenko et al. investigated pnicogen bonding in ammonium chloride (*P*-43 m) crystal using 120 K high-resolution (sinθ/λ = 1.2 Å^−1^) X-ray diffraction data [79]. They observed four weak σ-hole bonds in tetrahedral ammonium cation (NH_4_^+^) with Cl^−^ anion. Topological analysis of the experimental electron density reveals ρ (~0.05 eÅ^−3^) and Laplacian values similar to pnicogen bonding observed in molecular crystals (Figure 14).

**Figure 15:**
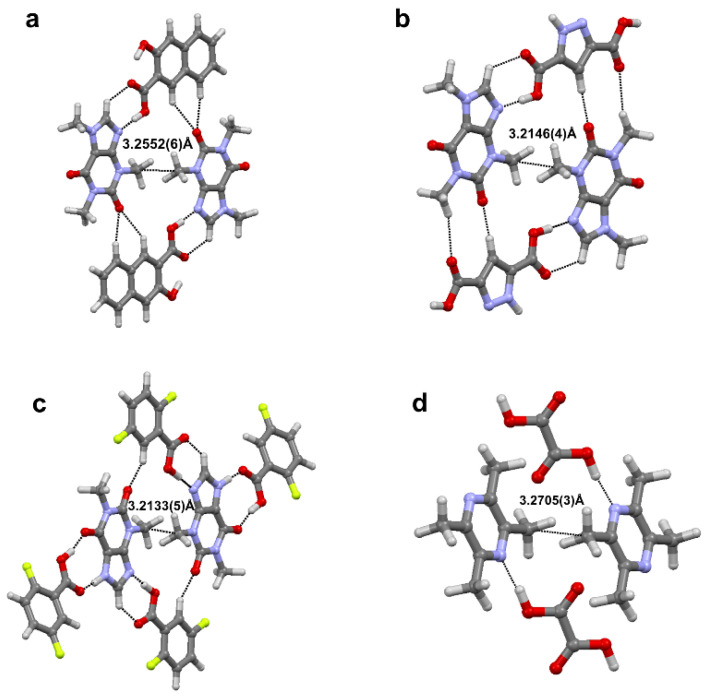
Me∙∙∙Me HI motif in the four cocrystals. (**a**) Caffeine:3-hydroxy-2-naphthoic acid (Caff-3HNA), (**b**) caffeine:3,5-pyrazoledicarboxylic acid (Caff-PZCA), (**c**) theophylline:2,5-diflurobenzoic acid (Theo-25DFBZA) and (**d**) 2,3,5,6-tetramethylpyrazine: oxalic acid (TMP-OA).

Aside from conventional σ-hole based weak interactions, we have investigated hydrophobic interaction (HI) between homopolar alkyl groups, e.g., methyl∙∙∙methyl (Me∙∙∙Me) interactions in molecular crystals [78]. HI, often explored in the solution-state aggregation of molecules, is the attractive force that induces the aggregation of nonpolar moieties in an aqueous medium [81]. HI are known to play a significant role in enzyme-substrate/drug-receptor binding [82] and the formation of micelles [83] and lipid bilayer in cell walls [84]. It is interesting to note that crystal structures of simple molecules such as propane, dimethylamine, and several drug molecules (*fenofibrate*, *wortmannin*, *etc*.), contain short Me∙∙∙Me contacts. Additionally, Protein Data Bank (PDB) contains more than 3000 crystal structures with Me∙∙∙Me HI interaction which indicates its ubiquity. Further, a detailed search in CSD with C∙∙∙C intermolecular distance less than the sum of van der Waals radii of C atoms (3.4 Å), resulted in 3038 crystal structures (without the disorder) with Me∙∙∙Me HI motifs. This motivated us to explore the electronic nature and importance of HIs in the solid state. We carried out a systematic study on the electronic nature of Me∙∙∙Me hydrophobic interactions in a series of multi-component crystals of biologically active molecules such as caffeine:3-hydroxy-2-naphthoic acid (Caff-3HNA), caffeine:3,5-pyrazoledicarboxylic acid (Caff-PZCA), theophylline:2,5-diflurobenzoic acid (Theo-25DFBZA) and 2,3,5,6-tetramethylpyrazine: oxalic acid (TMP-OA) using high resolution X-ray experimental charge density multipole modelling (CDMM). Figure 15 below shows various intermolecular interactions noted in these molecular pairs.

**Figure 16:**
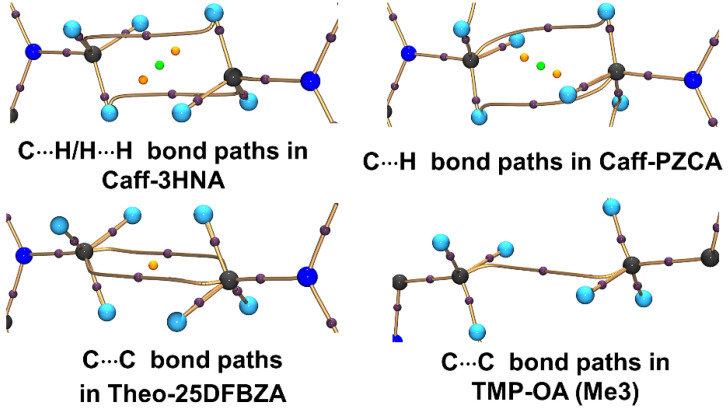
Bond paths (golden curved segments) along with critical points of hydrophobic Me∙∙∙Me interactions derived from experimental CDMM. (3,−1) BCPs, (3,+1) ring critical point (RCP), and (3,+3) cage critical points (CCP) are represented by the violet, orange, and green spheres. Reprinted with permission from reference. Ref. [78] Copyright 2019, American Chemical Society.

Quantitative analysis of the experimental EDs using QTAIM shows the presence of ED bond paths along with (3,−1) BCPs between the constituting atoms of Me groups thereby establishing the existence of corresponding Me∙∙∙Me interaction motifs (Figure 16). Interestingly, the observed bond path profiles connecting the two Me groups are inconsistent in different cases. These findings defy the concept of specific atom∙∙∙atom interactions in the HI regions. On the contrary, collective participation of constituting atoms of Me groups are observed that form different kinds of bond paths, i.e., both homonuclear and heteronuclear intermolecular bond paths-C∙∙∙H, H∙∙∙H, C∙∙∙C. Hence, HIs are essentially group∙∙∙group interactions as opposed to any conventional sigma-hole bonding or donor-acceptor interactions [19]. The values of rho, Laplacian, local kinetic energy densities (G), and the ratio of potential to kinetic energy densities (|V|/G) at BCPs are comparable to those of other weak noncovalent interactions discussed earlier in this review (Table 5).

**Figure 17:**
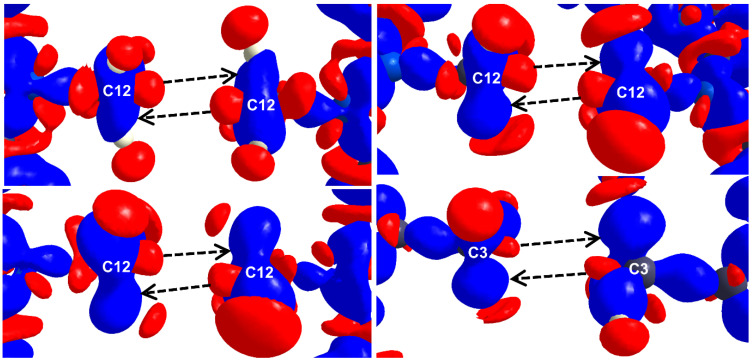
Experimental 3D deformation density maps for Caff-3HNA (**top left**), Caff-PZCA (**top right**), Theo-25DFBZA (**bottom left**), TMP-OA (Me3) (**bottom right**) plotted at a contour level of 0.08 eÅ^−3^. The blue region indicates charge concentration regions while red region indicates charge depleted regions, respectively. Reprinted with permission from reference. Ref. [78] Copyright 2019, American Chemical Society.

The experimental 3D static deformation maps reveal interesting details about Me∙∙∙Me HIs. (Figure 17). σ-holes on respective C centers (red lobes) along the C-X bond are directed towards each other as seen from the deformation maps. A closer look into the deformation maps suggests a slight “misalignment” in the relative orientation of the sigma holes. To rephrase it, the pair of X-C∙∙∙C-X bonds are not collinear in the intermolecular space. As a consequence, δ^+^ sigma holes are partially exposed to electron concentrated δ^−^ C-H bonding pairs (blue lumps as indicated by the black arrows). This possibly reduces the electrostatic repulsion between the Me groups.

In this context, it is worth mentioning that an offset interaction between the two methyl groups leading to C-H∙∙∙H-C dihydrogen interactions could be repulsive in nature, as shown by Thomas, Spackman, and co-workers [85]. They showed with the help of an X-ray charge density model that these interactions mode could be repulsive despite showing an interaction bond path in the topological analysis. In addition, the slightly repulsive C-H∙∙∙H-C dihydrogen interactions were shown to be playing a role in the plastic bending behavior of this crystal.

### 2.4. π-Holes Interactions

The description of π-holes originated from the concept of σ-holes which have similar properties. The depleted electron density regions perpendicular to portions of a molecular framework are characterized as π-holes. The π-holes can form with or without covalent π-bonds and they could be found in π non-conjugated or π-conjugated (aromatic or non-aromatic) organic molecules and non-conjugated inorganic molecules, eg., SO_2_, SeO_2_, BX_3_ (X = F, Cl, Br and I), silenes, etc. [86,87]. It was shown that the positive electrostatic potentials above carbonyl carbon atoms in acetyl fluoride and acetamide correlate well with their relative tendencies towards nucleophilic substitution reactions, such as hydrolysis [80]. Both positive σ-holes and π-holes can be present in one molecule and their interactions with negative sites such as lone pairs and anions are highly directional [53,88,89].

**Figure 18:**
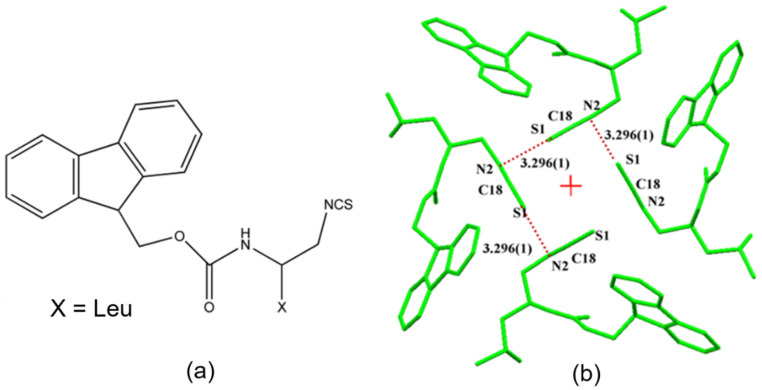
(**a**) Structural formula of F_moc_-Leu-ψ[CH_2_NCS], (**b**) N=C=S∙∙∙N=C=S interaction motif present in the low temperature (100K) crystal form. ‘+’ in red represents the 4_1_ screw axis. (Figure reproduced from reference [90]).

**Figure 19:**
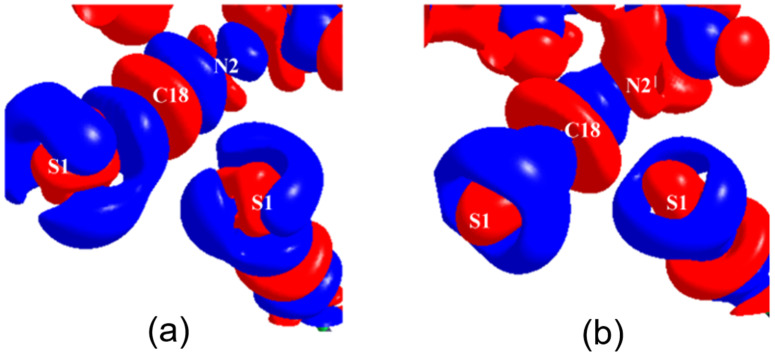
The 3D deformation density plots (**a**) experimental and (**b**) theoretical charge density analysis highlighting the N=C=S∙∙∙N=C=S interaction motif. Blue and red: +ve and –ve electron density, respectively. The deformation density contours are drawn at −0.05 eÅ^−3^. (Figure reproduced from reference [90]).

**Figure 20:**
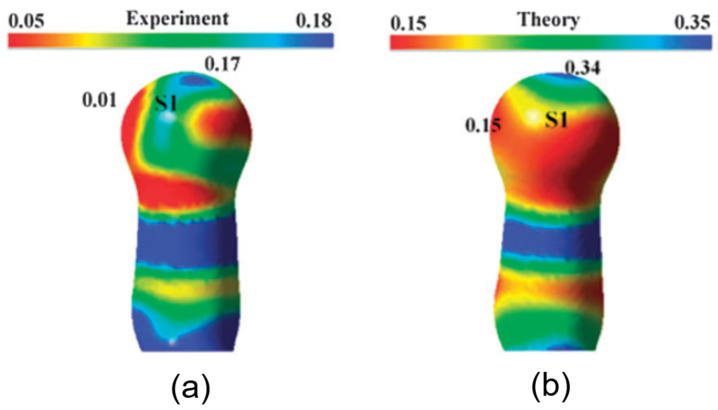
Electrostatic potential mapped on 0.5 eÅ^−3^ isodensity surface; (**a**) highlights a σ-hole on S (blue) along the extension of the N=C=S bond, (**b**) highlights a π-hole (blue) perpendicular to the C=N bond. Blue and red colours: +ve (more electropositive) and –ve regions (less electropositive), respectively. (Figure reproduced from reference [90]).

The simultaneous presence of σ-hole and π-hole was observed in the crystals of F_moc_-Leu-ψ[CH_2_NCS] (Figure 18a) [90]. It exhibited a temperature-induced reversible isomorphous phase transition and the low-temperature form at 100 K displayed a unique short N=C=S∙∙∙N=C=S intermolecular interaction (Figure 18b) which was characterized by experimental and theoretical charge density analysis as a stabilizing interaction involving both σ-holes and π-holes acting cooperatively. The σ-hole was identified on S along the extension of the N=C=S covalent bond and the π-hole was formed perpendicular to the CN bond as revealed in the 3D deformation density maps (Figure 19) and electrostatic potential maps (Figure 20).

## 3. Applications of Charge Density Analysis in Crystal Engineering and Pharmaceutical Sciences

We have demonstrated that the charge density topological features at the intermolecular interaction regions of the supramolecular synthons (certain recurring interaction motifs which act as supramolecular recognition units) are roughly conserved, at least in the examples we analyzed [91]. In earlier work, Munshi and Row classified hydrogen bonds into weak and strong classes on a quantitative scale based on the topological features of experimental and theoretical charge density models [92]. The charge density studies on the C-H∙∙∙F, C-H∙∙∙Cl, and C-H∙∙∙Br hydrogen bonds, which were considered to be in the weak regime of interactions, revealed the strength and significance of these new classes of hydrogen bonds. Notably, we demonstrated the strength of the trifurcated C-H∙∙∙O hydrogen bond motifs [93] which can even match or overcome the strength of a classical O-H∙∙∙O hydrogen-bonded motif via charge density derived interaction descriptors, thus providing quantitative evidence for the supramolecular co-operative effect of weak interactions (often referred to as the “Gulliver effect”). Within the context of crystal engineering, Lyssenko et al. attempted to rationalize the anomaly in the densities and the relative stabilities of the two commonly known polymorphs of *paracetamol* using charge-density derived descriptors [94], in terms of the strength of H-bonds in the higher stability of polymorph I, and relatively weaker H-bonds for the higher density polymorph II. In an attempt to quantify the lattice energies of polymorphs, Farrugia and co-workers used X-ray charge density derived interaction energy sums of *sulfathiazole* polymorphs. Their study demonstrated that the energy values from experimental multipole populations are heavily dependent on the refinement models, revealing a serious limitation of the technique. A more widely employed energy descriptor in quantifying interactions is the hydrogen bond energy formula obtained by Espinosa et al., an empirical relationship between interaction energies of a hydrogen bond (E_HB_) and the potential energy density at the bond critical points of the electron density bond paths [95]. However, a comparative study by Spackman has critically reviewed this method and cautioned of the caveats of employing the EML correlation to estimate interaction strengths [96]. For more insights into the applications of charge density studies in crystal engineering, the readers are directed to a recent review by Krawczuk and Macchi [97]. Some representative examples are discussed in this section.

Charge density studies contribute to pharmaceutical research in two major ways namely, the study of high-resolution structural features of drug molecules, i.e., active pharmaceutical ingredients (APIs), and understanding minute details of drug–protein interactions providing insights into rational drug design. A comprehensive review by Dittrich and Matta highlights the importance of charge density studies in a variety of applications in Medicinal Chemistry [98]. Some of the notable examples are discussed below.

### 3.1. Charge Density Studies of Pharmaceutical Compounds (APIs)

Experimental charge density distribution studies have unraveled several interesting aspects of pharmaceutically important molecules. Topological properties, such as reactive surface (zero Laplacian function) and variation in the electrostatic potential of the molecule derived from electron density distribution (EDD) analysis have been helpful in identifying the most preferred regions for intermolecular interactions. Findings from EDD experiments are often compared with high-level theoretical gas-phase calculations to find out the effects of periodic arrangements of molecules in the crystalline environment on the electronic configuration of the API. In most cases, the underlying non-covalent interactions provide specific attributes for the observed or predicted the phenomenon of crystal effects. An antithrombotic agent, *Terbogrel* [88], a neurotransmitter Taurine 2-aminoethane sulfonic acid [89] an angiotensin II receptor antagonist, *LR-B/081* [99,100], and an anti-TB drug molecule, *Pyrazinamide* [101] are among some of the earliest drug candidates studied by high-resolution X-ray crystallography. A detailed analysis of topological properties and ESP indicated preferred sites of intermolecular interactions, and the covalent, ionic, and non-covalent nature of interactions, along with the influence of the crystal effect. These studies highlighted specialty applications of EDD methodology in determining relative strengths of various intermolecular interactions present in the crystal, rational for stabilization of a particular molecular conformation through quantitative estimation of different intramolecular interactions. Investigation of electrostatic nature of interactions and estimation of dipole moments of molecules/molecular fragments, assessment of charge depletion and charge concentration regions of the molecule, etc. All intra- and intermolecular chemical bonding features (covalent and non-covalent) can be quantitatively described by the topological analysis of EDD. Key pharmacophoric features that are responsible for performance of the molecule as a drug are estimated through attractive electrostatic interactions. The overall structure may be deconvoluted highlighting importance of essential groups that provide significant energetic contributions towards binding energy and hence are critical for the biological activity of the drug. In general, electrostatic potential *ϕ*(*r*) maps of the molecule derived from the experimental EDD display signatures that provide hints towards probable drug-receptor recognition and other pharmaceutically important attributes of APIs. Some of the specific applications of EDD that are directly relevant to pharmaceutical science are discussed below.

Comparative studies on the experimentally derived EDD of a series of related compounds often provide important clues for structure–activity correlations. A study by Wagner et al. [102] on the two related penicillin derivatives, the active *penamecillin* as well as the inactive *penamecillin-1β-sulfoxide* provided insights into structure–activity relationships with respect to submolecular features. Importantly, the activity differences between these two molecules seemed to be not due to the difference in cleavage of the amide bond of the β-lactam ring, a feature generally perceived of extreme importance in explaining the mechanism of action of penicillins. The strength of this bond was found to be equal in both compounds as revealed by the topological analysis, therefore ruling out the correlation of bond strength on the drug activity of the molecule. Importantly, the two analogs were shown to be significantly different in their experimental electrostatic potentials, which may be attributed to their respective activity profiles. In a comparative study by Zhurova et al. [103], the steroidal estrogens were explored by experimental EDD determinations showing a correlation between the electronic properties of the molecules and their biological function. The relative binding affinities for four different conformers of *17α-Estradiol* and its chemical analog *17β-estradiol* were estimated using the observed electrostatic potentials. Grabowsky et al. demonstrated application of experimental EDD studies in lead optimization during rational drug design by examining three potential protease inhibitors (*aziridine*, *oxirane*, and acceptor-substituted olefin), known to facilitate inhibition of the proteases’ active sites through covalent bonding with the nucleophilic amino acids [104]. Collectively, through EDD-derived interaction energies and electrostatic potentials, it was shown that the *aziridine* analog was the most suitable for drug design toward the target protein. In addition, the charge density analysis also indicated a regioselective nucleophilic attack for this derivative and even provided hints about the reaction’s stereoselectivity. A high-resolution study of a series of anion receptor complexes of urea derivatives carried out by Kirby et al. demonstrated the utility of experimental EDD to understand different host–guest systems [105]. These multi-component systems were studied to quantify different N–H∙∙∙anion interactions and to deduce the contributions of respective intermolecular bonds to the resultant receptor anion attraction. One of the major outcomes of this study was a realization that the standard geometric criteria for intermolecular interactions may not necessarily always be followed, especially in cases of weak intermolecular interactions. The presence of such weaker interactions could only be ascertained by experimental EDD studies.

#### 3.1.1. Insights on Polymorphism and Relative Stability of Polymorphs

Accurate electron density measurements on the polymorphs of API can provide insights into electronic differences at the subatomic levels which may be associated with observed differences or properties. For example, studies on a pair of conformational polymorphs, namely A and B of anti-ulcer drug *famotidine* [106] showed striking similarities among electronic as well as electrostatic features of the two conformers in the respective polymorphs without any major differences in the interatomic interactions or in the atomic charges. In addition, similarities in the derived properties of the polymorphs, e.g., molecular dipole moment was noted. However, the differences between the polymorphs A and B in the ESP mapped on the molecular isodensity surface unraveled the variations between the two polymorphs. Interestingly, both the conformer had comparable areas of electronegative as well as electropositive regions; however, the average ESP in the electronegative region of polymorph A was found to be −40 kJ mol^−1^, while it was found to be −55 kJ mol^−1^ in case of polymorph B. Corroborating observations of molecular shape and dimensions in the two polymorphs, the EDD study concluded that the polymorphs may have different binding affinities and hence activities towards the target receptor site. Another interesting and outstanding example of the application of EDD study is in the characterization of the polymorphs of a classical analgesic drug molecule, *paracetamol* [94]. As mentioned earlier, the exceptional behavior of *paracetamol* polymorphs suggesting that “Higher density does not mean higher stability” was unraveled by the EDD study. It was demonstrated that the higher stability of a low-density form (polymorph I) over a higher density form (polymorph II) is dictated by the presence of stronger H-bonds in polymorph I. This resolved the debate on density-based relative stabilities of the two *paracetamol* polymorphs unambiguously. An unusual case of “hybridization induced polymorphism” of diuretic drug *acetazolamide* was rationalized by systematic experimental charge density studies on polymorphs I and II [63]. The change in electronic configuration features on the nitrogen atom (sp^3^ vs. sp^2^ hybridization state) and concomitant changes in the adjacent S-N covalent bond were found to be responsible for the occurrence of the so-called kinetic form, i.e., form II.

#### 3.1.2. Predicting Chemical Reactivity/Mechanism of Action of APIs

Reactivity and mechanism of action of APIs are often related to the nature of certain chemical bonds in the molecules. Charge density study is one of the very few techniques wherein such hypotheses can be validated experimentally by the visualization and quantification of various interatomic bonding features. Thomas et al., in their pioneering work on a series of organoselenium drug candidates, *ebeselan*, and its analogs, uncovered the mechanism of action of this class of antioxidant drug molecules through systematic analysis of intra- and intermolecular bonding features using the EDD approach [56]. A direct correlation between the intermolecular Se∙∙∙O chalcogen bond and electron density at the Se-N covalent bond further supported the mechanism of action of the drug which ultimately involves breakage of the Se-N bond.

#### 3.1.3. Applications in Formulation Development

The physicochemical properties of an API play an important role in its formulation as a viable drug product. Ghermani et al. explored the feasibility of a novel cyclodextrin-based formulation of a classical drug *Busulfan* through an experimental charge density study [107]. With the help of observed electrostatic features of the molecule, authors were able to explain the high crystallizability of the drug—a factor hindering the development of liquid phase (liposomal) formulations based on drug encapsulation through the liposomes. The high propensity of crystallization was attributed to the presence of terminal methylsulfonate groups due to its strong polar character. Additionally, the electrophilic carbon chain was indicated to stabilize association with β-cyclodextrin corroborating the viability of experimentally obtained *busulfan*/β-cyclodextrin formulation.

#### 3.1.4. Investigations on Exotic Non-Covalent Interactions in APIs

Revealing and validating the nature of interatomic interactions is among the prime applications of experimental EDD studies. In pharmaceuticals, with increasing complexities of new drug candidates, a variety of intermolecular interactions are manifested their solid forms. Sometimes, a detailed study of interactions, beyond classical hydrogen bonds, is necessary to explain the observed physicochemical properties of the molecules. Zhurova et al. demonstrated the existence of intermolecular hydrogen–hydrogen (H∙∙∙H) bonding for the first time in a steroid molecule, *Estrone* [108]. In another interesting study [109], it was confirmed that some of the strong intermolecular O∙∙∙H-O H-bonds in a nonsteroidal phytoestrogen, *genistein* possess a partial covalent character (incipient hydrogen bonds).

#### 3.1.5. Salt vs. Cocrystal Nature

Salts of APIs have been classically used for enhancement of bioavailability, stability as well as physical properties (density, flowability, etc.). In recent years, cocrystals are emerging as one of the alternative approaches as modified drug substance candidates. The major characteristic difference between salt and a cocrystal is with respect to the transfer of proton between a salt former/coformer and an API. While this difference can be identified using routine crystal structure determination, the differential becomes difficult for borderline cases. Hathwar et al. demonstrated the application of charge density analysis in clearly distinguishing a salt vs. a cocrystal in terms of analysis of EDD features in the intermolecular region of proton transfer [110]. The authors proposed a methodology for the quantification and verification of the cocrystal to salt continuum by assessing the interaction energies, integrated atomic charges, and other topological indicators. This approach is independent of any assumptions, (e.g., ∆pKa rule) and provides a fool-proof mechanism to assign “Salt” or “Cocrystal” nature to a molecular complex based on bond properties as per QTAIM considerations.

### 3.2. Charge Density Studies to Understand Protein-Ligand Interactions

Structural problems in medicinal chemistry can be tackled in an excellent way by combining CD studies and protein crystallography to address biological processes at the molecular and sub-molecular levels. While macromolecules (proteins or protein–ligand complexes) are generally not amenable to classical CD work due to experimental limitations and various structural dynamics and disorders, the availability of various multiple model databases comes to help. With such transferable models, macromolecular systems with normal data resolution and quality can be studied therefore bringing large molecule structures (proteins, DNA, and many other biological molecules) within the reach of CD research [111,112,113]. The CD properties in the interaction region of interest can be studied for such systems and intramolecular interaction energies can be calculated from the aspherical atoms refinement model [114]. This provides a fair idea of the reorganization of electron density of a drug molecule while associated with the active site and hence helps in understanding drug–receptor interactions in a quantitative manner.

The pioneering work on aldose reductase [115] was among the breakthroughs in the field. This was the first example wherein an enzyme of a significantly larger size, crystallized with an inhibitor, was studied at a significantly higher resolution, employing the charge density modeling where atoms are refined as aspherical moieties. Suspected electrostatic complementarity was confirmed in the system [116,117]. Dominiak et al. studied a series of high-resolution structures where derivatives of sialic acid and other inhibitor compounds were crystallized with influenza neuraminidases and successfully quantified various intermolecular interactions analyzed using charge density analysis. [118] Malińska et al. studied structures of *sunitinib malate* as well as molecular complexes of *sunitinib* cocrystallized with several protein kinases. [119] EDD study coupled with Hirshfeld surface analysis brought out similarities in interaction modes in the *sunitinib malate* structure and that at the active sites in the drug-enzyme cocrystals. Precisely, nine bond paths corresponding to various intermolecular interactions were found to be preserved in the API which was also observed in the API-kinase crystal structures. Interestingly, this study confirmed that *sunitinib* develops attractive interaction with different kinases with a comparable electrostatic driving factor and adjusts the molecular conformation that suites the binding site so as to enhance the electrostatically driven non-covalent interactions/H-bonds drug–receptor complexes. This attribute of the drug *sunitinib* explained its activity as a broad-spectrum kinase inhibitor. Additionally, studies on mid-sized peptide antibiotics *trichotoxin A50E* [120] and *thiostrepton* [121] are some notable examples in this field.

Despite several promising reports that assure wide applications of EDD estimations for drug–receptor complexes [112,113,114,115,116,117,118,119,120,121,122,123], the challenge of obtaining highly accurate datasets remains to be a major problem in the crystallographic studies of large molecules. While the more commonly encountered challenge is data resolution, the issues with positional inaccuracy of hydrogen and other atoms mainly due to the disorder of various components of the structure such as side chains, water molecules, and solvent molecules make the overall modeling exercise very difficult and sometimes erroneous. The derived properties need to be critically evaluated and validated as the correctness and reliability of parameters, e.g., estimated interaction energy values are highly dependent on the accuracy of the refined model.

## 4. Summary and Outlook

In summary, the studies on weak non-covalent interactions presented here demonstrate the power and limits of experimental charge density analysis by X-ray diffraction using the multipole modeling (CDMM) approach. Characterizing new types of intermolecular interactions using CDMM-derived descriptors could be useful in quantitatively classifying them based on their strengths and bonding features. Here, we have attempted to demonstrate how such studies could contribute to the field of crystal engineering and in the context of pharmaceutical drugs. In general, for organic molecular crystals, a reasonable agreement is observed between experimental and theoretical CDMM and derived properties. Extensive theoretical studies on a broad spectrum of NCIs by several computational research groups have helped understand, identify and classify them in terms of their energies, electron density features, and electrostatic origin. The research contributions from Politzer et al. [86] Frontera et al. [124] and Scheiner [125] is particularly significant in this context. While most computational studies focus on pairwise interactions of molecular dimers (“gas phase” dimers), X-ray charge density analysis offers the means to experimentally visualize and quantify them in crystalline environments. As a result of the combined research outputs from experimental CDMMs, it is now well established that for most small molecule organic crystals, computational estimates of molecular electronic properties might be a sufficient substitute for experimental charge density models—except in cases of unusual bonding or NCIs. However, this is not yet the case with crystalline materials containing heavy elements such as iodine. This poses challenges in accurately characterizing interactions involving heavy atoms (for example the robust halogen bonding interactions such as I∙∙∙N or I∙∙∙O). With the advent of quantum crystallography, including a variety of techniques such as Hirshfeld Atom Refinement (HAR), X-ray wavefunction refinement (XWR), along with libraries of extremely localized molecular orbitals (HAR-ELMO), experimental electron density studies might emerge into newer and wider applications in the field of non-covalent interactions.

## Figures and Tables

**Table 1:** The topological properties of the ED determined from the experimental multipolar model for Cl∙∙∙Cl interactions. Theoretical values from the *CRYSTAL* calculations are given in italics. *R*_ij_ = X_1_···X_2_, θ_1_ = C–X_1_···X_2_ and θ_2_ = X_1_···X_2_–C.

Interaction Geometry	R_ij_ (Å)	θ_1_/θ_2_ (°)	ρ_BCP_ eÅ^−3^	∇^2^ρ_BCP_ eÅ^−5^	ε	G(r_BCP_) (kJ mol^−1^ bohr^−3^)	V(r_BCP_) (kJ mol^−1^ bohr^−3^)	H(r_BCP_) (kJ mol^−1^ bohr^−3^)	$\frac{\left|V\right|}{G}$
Cl_1_∙∙∙Cl_1_ (*cis*)	3.3172(1)	158.7/158.7	0.05 0.06	0.66 0.72	0.02 0.03	13.5 15.4	−9.2 −11.2	4.3 4.2	0.68 0.73
Cl_1_∙∙∙Cl_1_ (*trans*)	3.5747(2)	150.6/150.6	0.03 0.04	0.41 0.44	0.11 0.11	7.8 9.1	−4.9 −6.1	2.9 3.0	0.62 0.67
Cl_1_∙∙∙Cl_1_ (*L*)	3.4668(2)	168.3/103.6	0.03 0.05	0.47 0.57	0.03 0.07	9.0 11.8	−5.6 −7.9	3.4 3.9	0.63 0.68

**Table 2:** Topological parameters from the experimental ED for noncovalent halogen interactions.

Interaction	*R*_ij_ (Å)	ρ (e Å^−3^)	∇^2^ρ (e Å^−5^)	V	|V|/G	Comment
Cl∙∙∙Cl	3.3172(1)	0.05	0.66	−10.2	0.73	Hathwar et al. [23]
Cl∙∙∙Cl	3.5747(2)	0.03	*0.41*	−5.5	0.66	Hathwar et al. [23]
Cl∙∙∙Cl	3.4668(2)	*0.03*	*0.47*	−6.1	0.64	Hathwar et al. [23]
Cl∙∙∙Cl	3.4343	0.06	0.6	−11.2	0.81	Bui et al. [26]
Cl∙∙∙Cl	3.4618	0.05	0.6	−9.7	0.74	Bui et al. [26]
Cl∙∙∙Cl	3.6129	0.04	0.5	−7.5	0.71	Bui et al. [26]
Cl∙∙∙F	3.0207(2)	0.05	0.84	−11.9	0.68	Hathwar et al. [32]
F∙∙∙F	2.8187(1)	0.04	0.82	−10.4	0.63	Hathwar et al. [32]
Cl∙∙∙O	3.0562(3)	0.05	0.80	−11.5	0.69	Hathwar et al. [33]
Br∙∙∙O	2.922	0.11	1.33	−27.9	0.87	Pavan et al. [34]
Br∙∙∙Br	3.6673	0.06	0.54	−10.6	0.84	Pavan et al. [35]
Br∙∙∙Cl	3.7327	0.04	0.40	−6.6	0.75	Pavan et al. [35]
Br∙∙∙Cl	3.6133	0.05	0.51	−8.9	0.78	Pavan et al. [35]
Cl∙∙∙Cl	2.9941	0.12	1.51	−32.0	0.87	Sarkar et al. [36]
Br∙∙∙Br	3.6673	0.05	0.46	−8.4	0.80	Pramanik et al. [37]
Br∙∙∙Cl	3.7327	0.04	0.43	−6.8	0.74	Pramanik et al. [37]
Br∙∙∙Cl	3.313	0.08	0.82	−16.7	0.86	Pramanik et al. [37]
Br∙∙∙Br	3.2324	0.06	0.67	−11.8	0.79	Pavan et al. [34]
Br∙∙∙Br	3.7098	0.04	0.51	−7.6	0.70	Pavan et al. [34]
F∙∙∙F	2.6627	0.06	1.3	−17.6	0.66	Pavan et al. [34]
F∙∙∙F	2.824	0.04	0.9	−11.1	0.62	Pavan et al. [34]
F∙∙∙F	2.8091	0.05	1.03	−13.6	0.65	Chopra et al. [38]
F∙∙∙F	2.569	0.07	0.93	−15.9	0.77	Chopra et al. [38]
Br∙∙∙O	2.7575	0.135	1.87	−39.2	0.87	Erakovic et al. [39]
Br∙∙∙N	2.3194(4)	0.379	3.63	−157.0	1.23	Erakovic et al. [39]
N∙∙∙I	2.6625	0.36	1.95	−131.6	1.42	Wang et al. [40]
Cl∙∙∙Cl	3.1912(6)	0.11	1.102	−25.8	0.92	Wang et al. [41]
I∙∙∙N	2.7804	0.24	1.96	−75.7	1.17	Bianchi et al. [42]
I∙∙∙O	2.7523	0.20	2.04	−61.3	1.05	Bianchi et al. [43]
I∙∙∙O	2.9824	0.10	1.307	−25.3	0.83	Wang et al. [44]
I∙∙∙I	2.789	0.40	2.02	−154.0	1.47	Nelyubina et al. [45]

**Table 4:** Topological parameters of intramolecular S∙∙∙O chalcogen bonds in a series of sulfa drugs obtained from experimental charge density studies. Table and Figure reproduced from Thomas et al. [62].

Molecule	*R*_ij_ for S∙∙∙O(Å)	ρ(eÅ^−3^)	∇^2^ρ (eÅ^−5^)	|V|/G	*2G/|V|*	*G*/ρ	Method
Sulfamethizole-sulfate [60]	2.816	0.13	1.50	0.92	2.18	0.74	MM
	2.753	0.15	1.60	0.97	2.06	0.72	
*Acetazolamide* (form I) [59]	2.752	0.14	0.42	1.41	1.42	0.36	XWR
*Acetazolamide* (form II) [63]	2.608	0.16	2.19	0.91	2.21	0.88	MM
Sulfathiazole Polymorphs [61] Ia,	2.9913	0.10	1.11	0.88	2.28	0.69	MM
Ib	2.9694	0.09	1.09	0.83	2.40	0.73	MM
II	2.9583	0.10	0.96	0.92	2.18	0.62	MM
IIIa	2.9382	0.10	1.11	0.88	2.28	0.69	MM
IIIb	2.8834	0.11	1.23	0.89	2.24	0.71	MM
IV	2.9006	0.11	1.16	0.91	2.20	0.68	MM

G = Kinetic energy density, V = potential energy density (au). MM = Multipole model, and XWR = X-ray wavefunction refinement.

**Table 5:** Experimental topological values of Me∙∙∙Me HIs at (3,−1) bond critical points.

Molecules	*R*_ij_ (Å)	ρ (eÅ^−3^)	∇^2^ρ (eÅ^−5^)	ε	G ^a^	|V|/G
Caff-3HNA	3.0453	0.047(4)	0.451(1)	1.02	10.1	0.81
Caff-PZCA	3.0893	0.037(4)	0.409(2)	2.68	8.7	0.75
Theo-25DFBZA	3.3037	0.009(4)	0.434(1)	1.51	8.0	0.53
TMP-OA	3.2694	0.037(3)	0.598(1)	0.64	12.1	0.68

^a^ Kinetic energy density (G) is in kJ mol^−1^ au^−1^.

## Abbreviations

ADP	Anisotropic displacement parameter
AIM	Atoms in molecules
API	Active pharmaceutical ingredient
BCP	Bond critical point
CC	Charge concentration
CD	Charge depletion
CD	Charge density
CDMM	Charge density multipole modeling
CSD	Cambridge structural database
ED	Electron density
EDD	Electron density distribution
ELMO	Extremely localized molecular orbitals
ESP	Electrostatic potential
FTIR	Fourier transform infrared
HI	Hydrophobic interaction
HAR	Hirshfeld Atom Refinement
IUCr	International Union of Crystallography
IUPAC	International union of pure and applied chemistry
LUMO	Lowest unoccupied molecular orbital
MESP	Molecular electrostatic potential
NCI	Non-covalent interactions
NOESY	Nuclear overhauser effect spectroscopy
PDB	Protein data bank
QTAIM	Quantum theory of atoms in molecules
RCP	Ring critical points
VSCC	Valence shell charge concentration
XB	Halogen bond
XWR	X-ray wavefunction refinement
